# In Vitro Infection with Hepatitis B Virus Using Differentiated Human Serum Culture of Huh7.5-NTCP Cells without Requiring Dimethyl Sulfoxide

**DOI:** 10.3390/v13010097

**Published:** 2021-01-12

**Authors:** Connie Le, Reshma Sirajee, Rineke Steenbergen, Michael A. Joyce, William R. Addison, D. Lorne Tyrrell

**Affiliations:** Li Ka Shing Institute of Virology, Department of Medical Microbiology and Immunology, 6010 Katz Centre for Health Research, University of Alberta, Edmonton, AB T6G 2E1, Canada; cle1@ualberta.ca (C.L.); rsirajee@ualberta.ca (R.S.); rineke@ualberta.ca (R.S.); maj2@ualberta.ca (M.A.J.); bill.addison@ualberta.ca (W.R.A.)

**Keywords:** hepatitis B virus (HBV), hepatoma cell culture, sodium taurocholate co-transporting polypeptide (NTCP), differentiated Huh7.5-NTCP human serum culture, dimethyl sulfoxide (DMSO)

## Abstract

An estimated two billion people worldwide have been infected with hepatitis B virus (HBV). Despite the high infectivity of HBV in vivo, a lack of easily infectable in vitro culture systems hinders studies of HBV. Overexpression of the sodium taurocholate co-transporting polypeptide (NTCP) bile acid transporter in hepatoma cells improved infection efficiency. We report here a hepatoma cell culture system that does not require dimethyl sulfoxide (DMSO) for HBV infection. We overexpressed NTCP in Huh7.5 cells and allowed these cells to differentiate in a medium supplemented with human serum (HS) instead of fetal bovine serum (FBS). We show that human serum culture enhanced HBV infection in Huh7.5-NTCP cells, e.g., in HS cultures, HBV pgRNA levels were increased by as much as 200-fold in comparison with FBS cultures and 19-fold in comparison with FBS+DMSO cultures. Human serum culture increased levels of hepatocyte differentiation markers, such as albumin secretion, in Huh7.5-NTCP cells to similar levels found in primary human hepatocytes. N-glycosylation of NTCP induced by culture in human serum may contribute to viral entry. Our study demonstrates an in vitro HBV infection of Huh7.5-NTCP cells without the use of potentially toxic DMSO.

## 1. Introduction

Hepatitis B virus (HBV) represents an enormous public health burden with an estimated two billion individuals worldwide having been infected with the virus, resulting in 250–300 million people chronically carrying the infection [1,2,3,4,5]. HBV chronic carriers are at high risk of developing severe liver diseases, such as cirrhosis and cancer, culminating in 887,000 HBV-associated deaths annually. Conventional nucleoside analogue therapy suppresses replication without fully clearing the virus; therefore, the therapy is lifelong and can pose a financial burden [4,5,6,7,8]. Despite the global impact of HBV and advances in therapeutics [9,10,11,12,13,14], a cure for this chronic infection is yet to be developed.

HBV infection in immortalized liver cells is generally inefficient compared to HBV infection in the liver [15,16,17,18]. A major obstacle to studies of HBV has been the lack of an easily infectable cell culture system [16,17,18,19,20,21]. HBV-infectable primary human hepatocytes are expensive, difficult to obtain, rapidly de-differentiate ex vivo, and can only survive for a few weeks in culture [22,23,24]. Until recently, HepaRG cells were the only immortalized hepatoma cell line that could be infected with HBV, but had a low percentage of successful infection [25,26]. Other hepatoma cell lines cannot be infected with HBV, but can be transfected with HBV expression plasmids and, having bypassed cell entry, proceed with HBV infection from the genome transcription step [27,28]. Some cell lines, such as HepAD38 and HepG2.2.15, have integrated HBV genomes, which also recapitulate infection from the point of genome transcription to the release of infectious virus [29,30]. These systems permit investigations into post-transcriptional stages of infection.

Yan et al. demonstrated high-affinity binding between the HBV pre-S1 envelope protein and the sodium taurocholate cotransporting polypeptide (NTCP) bile acid transporter [31]. Furthermore, overexpression of NTCP renders otherwise unsusceptible hepatoma cells permissive to HBV infection. This significant discovery of a putative HBV entry receptor has benefitted the decades-long search for an easy-to-maintain cell culture system that supports the entire HBV lifecycle. This culture system, requiring the use of 2–2.5% dimethyl sulfoxide (DMSO), is now widely used to study HBV [31,32,33,34,35,36,37,38,39,40,41,42].

Our group has shown that culturing the human hepatoma cell line Huh7 or Huh7.5 in a medium supplemented with human serum (HS) increases production of hepatitis C virus (HCV) [43]. Cells cultured in an HS-supplemented medium underwent growth arrest and developed characteristics similar to primary human hepatocytes, including a cuboid morphology, formation of bile canalicular surfaces, restored lipid metabolism, contact inhibition, differentiation marker expression, reversal of the Warburg effect, very low-density lipoprotein (VLDL) secretion, and increased expression of cytochrome P450 [44,45,46]. This method of producing hepatocyte-like cells enhanced production of HCV 1000-fold and resulted in a virus that more closely resembled the HCV present in the serum of infected patients.

Previous studies showed that overexpression of NTCP in hepatoma cells only moderately improved infection efficiency and, following infection, these cultures must be maintained in high concentrations (2–2.5%) of DMSO for infection [31,32,33,34,35,36,37,38,39,40,41]. However, DMSO is known to cause a variety of adverse effects on cells, such as significant alterations in viability and protein expression [47,48,49,50,51]. Therefore, an HBV infection model that eliminates the requirement of DMSO treatment would be desirable to more closely mimic physiological conditions [52].

The primary objective of this research was to explore alternative cell culture models for HBV infection. We hypothesized that overexpression of NTCP in Huh7.5 cells and differentiation of these hepatoma cells in HS-containing media would enhance HBV infection. We report here that culture of Huh7.5-NTCP cells in human serum permitted robust HBV infection in the absence of DMSO.

## 2. Materials and Methods

### 2.1. Production of Lentiviral Vectors Expressing NTCP

NTCP-expressing lentiviral expression plasmid with a puromycin selectable marker was purchased from GeneCopoeia (Rockville, MD, USA). Lentiviral particles were generated in HEK-293T cells according to a previously reported method [53]. HEK-293T cells from American Type Culture Collection (ATCC, Manassas, VA, USA) were seeded at 50% confluence on poly-L-lysine-coated T150 flasks. Transfection was performed with Lipofectamine 2000 (Invitrogen, Carlsbad, CA, USA) according to manufacturer’s protocols.

### 2.2. Establishment of Stable NTCP-Expressing Huh7.5 Cell Line (Huh7.5-NTCP)

Huh7.5 cells, a kind gift from Dr C. Rice (Rockefeller University, New York, NY, USA), were maintained in Dulbecco’s modified Eagle’s medium (DMEM) (Sigma Aldrich, St. Louis, MO. D5796, high-glucose, with L-glutamine and sodium bicarbonate, without sodium pyruvate) supplemented with 10% fetal bovine serum (FBS) and penicillin/streptomycin. Briefly, low-passage Huh7.5 cells were seeded at 4 × 10^5^ cells per well of a 6-well plate. To the lentiviral stock, polybrene was added to 4 μg/mL and HEPES (pH 7.0) to 20 mM. The lentivirus inoculum (1 mL) was added to each well intended for transduction. The 6-well plate was then centrifuged at 150× *g* for 1 h at 37 °C. Following centrifugation, the lentivirus was further incubated with the cells for 6 h at 37 °C in 5% CO_2_. The medium for the transduced cells was then changed to DMEM containing 10% FBS. After 48 h of incubation at 37 °C, the medium was changed to DMEM containing 10% FBS and 0.1 μg/mL puromycin to select for cells that were successfully transduced. Transduced cells were cultured and selected in puromycin for one week prior to use in subsequent assays.

Overexpression of NTCP in transduced Huh7.5-NTCP cells was assessed and confirmed by RT-qPCR analysis of total RNA, flow cytometry analysis, and immunofluorescence staining of NTCP using the anti-NTCP antibody (Abcam, Cambridge, UK. ab175289). RT-qPCR was performed with the forward primer (5’-GGAGGGAACCTGTCCAATGTC-3′), reverse primer (5′-CATGCCAAGGGCACAGAAG-3′), and probe (5′-[6FAM]ACATGAACC/ZEN/TCAGCATTGTGATGACCACC-[IABk]-3′), all purchased from Integrated DNA Technologies (IDT, Coralville, IA). ΔΔCT values were calculated to determine fold change in NTCP mRNA expression. RT-qPCR for hypoxanthine-guanine phosphoribosyltransferase (HPRT) mRNA was performed using the Taqman primer probe mix from Applied Biosystems (Foster City, CA. cat No. 4326321E).

### 2.3. Conventional Culture of Huh7.5 Cells and Huh7.5-NTCP Cells

Huh7.5 and Huh7.5-NTCP cells were maintained in a DMEM medium supplemented with 10% FBS. These cells reached confluence within 3–4 days in culture and were re-seeded twice a week at 25% seeding density. When reseeding confluent cultures, cell monolayers were washed once with filter-sterilized PBS (136.9 mM NaCl, 2.68 mM KCl, 6.48 mM Na2HPO4, and 0.866 mM KH2PO4, pH 7.4). Subsequently, adherent cells were detached by adding ATV solution (107.3 mM KCl, 6.84 mM NaCl, 11.9 mM NaHCO3, 3.2 mM dextrose, 0.5 g/L trypsin, and 0.5 mM disodium EDTA) and incubating at 37 °C for 3 min. The flask was then gently percussed and the trypsin was inactivated by the addition of DMEM containing 10% FBS. Figure S1 shows the timeline for culturing and infecting cells in the different media.

### 2.4. Differentiation of Huh7.5 and Huh7.5-NTCP Cells in Human Serum

Confluent Huh7.5 and Huh7.5-NTCP cells, at passage 30 or fewer, were trypsinized with ATV solution and subsequently the trypsin was inactivated by the addition of DMEM supplemented with 4% pooled adult human serum (HS) (Valley Biomedical, Winchester, VA, USA). The cells were then plated in a DMEM containing 4% HS at a density of 30%. After 4 days, the cells were again trypsinized as described and plated at 50% density in a DMEM containing 4% HS. Culture medium was then changed twice a week for an additional 17 days for a total of 21 days. During this time, the cells underwent contact inhibition and differentiation.

### 2.5. Infection of Cells with HBV

HBV inoculum was prepared using HepAD38 cells, a hepatoma cell line with an integrated greater than genome length copy of HBV genotype D subtype ayw [30]. Huh7.5 and Huh7.5-NTCP cells were infected with HBV at a multiplicity of infection (MOI) of 500 genome equivalents, unless otherwise stated, in the presence of 4% PEG 8000 for 18 h at 37 ℃. Four culture media were compared in this study. DMEM medium was supplemented with: 10% FBS; 10% FBS and 2% DMSO; 4% HS; and 4% HS and 2% DMSO. After HBV infection, cells were washed three times with 1× PBS and then maintained in one of these four media. The medium was changed twice a week following infection (see Figure S1 for the timelines of culturing and infection).

### 2.6. Culture of PXB Cells

PXB cells were purchased from PhoenixBio (Hiroshima, Japan) and seeded at 1 × 10^7^ cells per 24-well plate as per the manufacturer’s recommendation. PXB cells are human hepatocytes isolated from chimeric humanized liver mice for the purposes of use for in vitro culture experiments [54]. The cells were cultured for seven days in a DMSO-supplemented hepatocyte clonal growth medium (dHCGM) purchased from PheonixBio. These human hepatocytes were then subjected to the same protocol as the Huh7.5-NTCP cells for assaying albumin secretion.

### 2.7. Analysis of Pregenomic RNA (pgRNA)

RNA was isolated from cell monolayers using either QIAzol reagent (Qiagen, Hilden, Germany) or Nucleospin RNA spin columns (Macherey Nagel, Duren, Germany) following the manufactures’ procedures. Pregenomic RNA was measured using qPCR using the forward primer (5′-GGAGTGTGGATTCGCACTCCT-3′), reverse primer (5′- AGATTGAGATCTTCTGCGAC-3′), and Taqman probe (5′-[6FAM]-AGGCAGGTCCCCTAGAAGAAGAACTCC-[BHQ1]-3′). The qPCR cycling conditions were 95 °C for 20 s, followed by 45 cycles of 95 °C for 1 s and then 60 °C for 20 s. The ramp speed between cycling steps was 4.14 °C per second. The results were expressed as pgRNA gene equivalents per 10 ng of total RNA.

### 2.8. Analysis of Covalently Closed Circular DNA (cccDNA)

Genomic DNA (gDNA) was isolated using a DNeasy Blood and Tissue kit (Qiagen) according to the manufacturer’s protocol. The culture supernatant was removed and cell monolayers were rinsed once with PBS. For cccDNA isolation, the other viral DNA species that contain gaps in the DNA and are not completely circular were digested by mixing 1 μg of cellular DNA with 25 units of exonuclease III (New England Biolabs, NEB, Ipswich, MA) and 1× final concentration of NEB buffer 1 (New England Biolabs). This mixture was then incubated at 37 °C for 1 h and then heat inactivated at 70 °C for 30 min.

HBV cccDNA was measured using qPCR using the forward primer (5′-CTCCCCGTCTGTGCCTTCT-3′), reverse primer (5′-GCCCCAAAGCCACCCAAG-3′), and Taqman probe (5′-[6FAM]-AGCGAAGTGCACACGGACCGGCAGA-[BHQ1]-3′). Quantitative PCR was performed using TaqMan Fast Advanced Master Mix, but the cycling protocol was modified to have longer incubations at cycling steps and slower ramp speeds to accommodate the large amplicon size and low amounts of cccDNA. The cccDNA samples were held at 95 °C for 10 min, followed by 50 cycles of 95 °C for 10 s, 62 °C for 10 s, and 72 °C for 20 s. The ramp speed between qPCR steps was 1.6 °C per second. The qPCR analysis was conducted on MicroAmp Fast Optical 96-well reaction plates (Applied Biosystems) and the QuantStudio 3 Real-Time PCR system (Applied Biosystems) [55]. Covalently closed circular DNA levels were expressed as cccDNA copies per 10 ng total gDNA. PCR analysis of mtDNA use the forward primer (5′-ACACTCATCGCCCTTACCAC-3′), reverse primer (5′-GTTGATGCAGAGTGGGGTTT-3′), and the SYBR Green probe.

### 2.9. Enzyme-Linked Immunosorbent Assays (ELISAs)

HBsAg was quantified using the QuickTiter Hepatitis B surface antigen (HBsAg) ELISA kit (Cell Biolabs Inc., San Diego, CA) according to the manufacturer’s protocol. Briefly, HBV virions in culture medium samples were inactivated by the addition of Triton X-100 to a final concentration of 0.5% and heating at 56 °C for 30 min. Recombinant HBsAg standards and samples were loaded onto the anti-HBsAg antibody-coated wells. The plate was covered and incubated at 37 °C for 2 h. The wells were washed 5 times with 1× buffer. The wells were then incubated with the FITC-conjugated anti-HBsAg monoclonal antibody (diluted 1:250 in the assay diluent) for 1 h at room temperature. The wells were once again washed 5 times with 1× wash buffer. The HRP-conjugated anti-FITC monoclonal antibody (diluted 1:1000 in the assay diluent) was added and incubated for 1 h at room temperature. After washing the wells 5 times, 100 μL of the substrate solution were added and incubated at room temperature for 15 min. After that, 100 μL of the stop solution were added to each well and absorbance at 450 nm was measured immediately using a spectrophotometer.

Secreted albumin was measured using sandwich ELISA as described previously [43]. Briefly, cells were washed extensively with serum-free DMEM and then once with serum-free OptiMEM. The last wash with OptiMEM was collected to assess background levels of albumin. Fresh serum-free OptiMEM was added to each well with samples collected 6 and 24 h afterwards. ELISA plates were coated with 100 μL of 0.625 μg/mL goat anti-human albumin antibody (Bethyl Laboratories, Montgomery, TX. A80229A) diluted in the coating buffer (50 mM NaHCO3, 51.9 mM Na_2_CO_3_) overnight at 4 °C. The next day, wells were washed three times with Tris-buffered saline + 0.1% Tween-20 (TBST) and incubated in the blocking buffer (TBST and 1% gelatin; Bio-Rad, Hercules, CA. 1706537) for 30 min at room temperature. Samples were incubated on the antibody-coated plate for 1 h at room temperature. The wells were then washed three times with TBST and incubated with 100 μL of 0.625 μg/mL goat anti-human albumin HRP-conjugated antibody (Bethyl, A80229P) diluted in TBST for 1 h at room temperature. The plate was washed three times with TBST and the wells were incubated with 100 μL of the TMB (3,3′,5,5′-tetramethylbenzidine) substrate for 15 min. Absorbance at 450 nm was measured with a Perkin Elmer Enspire 2300 plate reader after adding 100 μL of 1 M phosphoric acid.

### 2.10. Aptamer-Binding Assay for the E Antigen (HBeAg)

HBeAg was determined using a sandwich aptamer-binding assay (Figure S2A) as reported recently [56]. Briefly, the NH_2_-A-9S aptamer (10 pmol) in 1× PBS buffer (10 mM sodium phosphate, 137 mM NaCl, and 4.5 mM KCl, pH 7.4) was heated to 95 °C for 10 min and then cooled to 0 °C for 10 min before the addition of MgCl_2_ to a final concentration of 7 mM. The aptamer solution was then incubated at room temperature for 10 min. The refolded NH_2_-A-9S aptamer solution was added to an Immobilizer Amino 96-well plate. Incubation at room temperature for 6 h allowed for the conjugation of the NH_2_-A-9S aptamer to the surface of the 96-well plate. A binding buffer (1× BB) containing 50 mM Tris-HCl (pH 7.4), 5 mM KCl, 50 mM NaCl, 7 mM MgCl_2_, and 0.05% Tween 20 was added to the plate and incubated at room temperature for 30 min. The 96-well plate was ready for use after removal of the excess aptamer solution and buffer solution.

For the determination of HBeAg in each sample, triplicate aliquots of 100 µL pretreated sample were added into three wells. The 96-well plate was incubated at 37 °C for 2 h. The plate was washed five times with the washing buffer that contained 1× binding buffer and 0.1% casein. To each well, we added 100 µL of biotinylated eAg3-Py aptamer (1 µM) in 1× binding buffer supplemented with 0.5% BSA and 5 µM blocker sequence (TGGGC). The plate was incubated at 37 °C for 30 min and washed three times with the washing buffer. To each well, we added 100 μL of 50× diluted horseradish peroxidase (HRP)-conjugated streptavidin. The plate was incubated at room temperature for 30 min and each well was washed three times with the washing buffer. Finally, 100 μL of the substrate solution (Invitrogen) were added into each well and incubated for 30 min before the addition of 2 M phosphoric acid to stop the reaction of HRP. Absorbance at 450 nm was measured using a plate reader (Beckman, Indianapolis, IN, USA).

### 2.11. Immunofluorescence Staining of Huh7.5 Cells Overexpressing NTCP

For immunofluorescence staining of NTCP, Huh7.5 or Huh7.5-NTCP cells were seeded at 25% confluence onto glass coverslips placed in culture wells. The next day, cell monolayers were washed with PBS and then fixed with 4% formaldehyde in 1× PBS for 10 min at 37 °C. The formaldehyde solution was removed and coverslips were washed three times with PBS. Cells were permeabilized for 5 min with 0.1% Triton-X100 in PBS, then coverslips were washed with PBS. A block solution (1× PBS containing 5% BSA) was added and incubated for 1 h at room temperature. The cells were then incubated with rabbit anti-NTCP antibody (Abcam, ab175289; diluted 1:200 to a final concentration of 2.5 μg/mL in the block solution) at 4 °C overnight in a moist chamber. The coverslips were then washed three times with 1× PBS. Alexa568-labeled goat anti-rabbit secondary antibody (Invitrogen, A11036; diluted 1:400 (final concentration of 5 μg/mL) in 1× PBS with 5% BSA) was added and incubated for 1 h at room temperature. The coverslips were washed three times with 1× PBS before the addition of Hoechst 33,342 (Invitrogen) (diluted 1:5000 in 1× PBS). After a final PBS wash, Vectashield mounting medium for fluorescence (Vector Laboratories, Burlingame, CA. H-1000) was added. The cells were imaged with a Quorum Wave FX-2 spinning disk confocal microscope using 20×/0.85 NA oil immersion lenses and Velocity v. 6.2.1 software (Perkin Elmer, Waltham, MA).

Immunofluorescence staining of HBV-infected Huh7.5-NTCP cells was performed using procedures similar to those previously described [36]. After 14 days of infection, cells were fixed with formaldehyde and permeabilized with 0.1% Triton X-100 in PBS for 1 min at room temperature. Cell monolayers were washed three times with PBS after permeabilization and the plate was blocked with 1× PBS containing 5% BSA. The cells were then stained using rabbit anti-HBV core (Invitrogen, PA5-16368; diluted 1:200 in 1× PBS with 5% BSA) and Alexa568-conjugated goat anti-rabbit secondary antibody (Invitrogen, A11036; diluted 1:400 in 1× PBS with 5% BSA).

### 2.12. Flow Cytometry Analysis of NTCP Expression

Adherent cells were dissociated with accutase (Gibco, Dublin, Ireland. A1105-01), washed, and resuspended cells were blocked in 10% filtered human serum with 5% BSA in PBS. The cells were then analyzed using rabbit anti-NTCP primary antibody (Abcam, ab175289; diluted 1:100 (final concentration of 5 μg/mL) in the block solution) and the Alexa647-labeled anti-rabbit secondary antibody (Invitrogen, A31573; diluted 1:2000 (final concentration of 1 μg/mL) in the block solution). Flow cytometry was conducted on a BD LSR Fortessa X-20 instrument with BD FACSDIVA software (version 8.0.1) (BD Biosciences, San Jose, CA, USA).

### 2.13. Western Blotting

Cell monolayers were washed twice with PBS and then lysed on ice for 10 min using a radioimmunoprecipitation assay (RIPA) buffer (50 mM Tris-HCl, pH 8.0, 150 mM NaCl, 0.1% SDS, 1% Triton X-100, 0.5% deoxycholic acid in Milli-Q water) with the addition of EDTA-free protease inhibitor (Roche, Basel, Switzerland). This whole cell lysate was centrifuged at 18,000× *g* for 15 min and the supernatant was collected. The protein concentration in cell lysates was quantified with the micro bicinchoninic acid (BCA) protein assay using the manufacturer’s protocol (Pierce, Rockford, IL).

A 10% SDS-polyacrylamide gel of 1.5 mm thickness was used for gel electrophoresis separation. The denatured protein samples as well as the pre-stained protein standard ladder (Fisher Scientific, Waltham, MA) were run at an initial electrophoretic voltage of 80 V for 30 min, and then 160 V for approximately 1 h. The separated proteins were transferred onto a nitrocellulose membrane (Amersham Hybond-ECL, GE, Marlborough, MA). The membrane was blocked, washed, and incubated with the rabbit anti-NTCP antibody (Abcam, ab175289; diluted 1:1000) and mouse anti-tubulin antibody (diluted 1:3000). Licor IRDye goat anti-rabbit 680 and goat anti-mouse 800 secondary antibodies (Licor, Lincoln, NE. cat. No. 926-32221 and cat. No. 926-32210, respectively) were used to detect the proteins. The membrane was scanned using a Licor Odyssey CLx imaging system and the images were analyzed using Image Studio software (Licor, Lincoln, NE).

### 2.14. Nanoluciferase Reporter Luminescence Assay

Constructs for producing HBV virus containing the nanoluciferase (NL) reporter were a kind gift from. K. Shimotohno (Research Center for Hepatitis and Immunology, National Center for Global Health and Medicine, Tokyo, Japan) [57]. The HBV/NL plasmid, depicted in Figure S3, encodes the HBV genome with the nanoluciferase (NL) gene in frame with the viral pre-core/core open reading frame. This insertion of NL disrupts the pre-core/core and polymerase open reading frames, preventing expression of these proteins. The epsilon sequence, a stem loop secondary structure in pgRNA required for its encapsidation, remains intact in pgRNA from the HBV/NL plasmid. In contrast, the HBV-D plasmid encodes all of the HBV proteins; however, it contains sense mutations that disrupt the epsilon sequence secondary structure. Therefore, when the HBV/NL plasmid is co-transfected with the packaging-deficient HBV-D helper plasmid, the pgRNA generated by the HBV/NL plasmid is encapsidated with the core and polymerase proteins generated from the HBV-D plasmid. This creates non-replicative HBV/NL virions that express NL upon infection. Therefore, infection with recombinant HBV/NL virions generates NL activity, which is a surrogate marker for translation from viral RNA. Hence, the HBV/NL virus is a useful tool for assaying early steps in HBV infection, from entry to transcription.

Huh7.5-NTCP cells were infected with HBV/NL in the same manner as described for HBV infection. The Nano-Glo Luciferase Assay System (Promega, Madison, WI) was used according to the manufacturer’s protocol to assess nanoluciferase reporter activity. Briefly, for a 96-well plate with 100 μL medium per well, 50 μL of the medium were removed, leaving 50 μL in each well. Subsequently, 50 μL of the Nano-Glo reagent (a 1:50 solution of substrate/buffer) was added to each well and incubated for 2 min at room temperature. The contents of the wells were thoroughly mixed to lyse the cell monolayers and then the luminescence of 50 μL of the mixture was measured on a Perkin Elmer Enspire 2300 plate reader.

### 2.15. Statistical Analysis

Statistical analysis was performed using Prism software for Mac OS version 8 (GraphPad, San Diego, CA). All data are represented as mean values ± standard deviation. Experiments comparing two groups were analyzed using unpaired Student’s *t*-tests. One-way analysis of variance (ANOVA) with the Bonferroni correction for multiple comparisons was used to evaluate experiments with more than two groups. Two-way ANOVA with the Bonferroni correction for multiple comparisons was used to assess experiments with two independent variables. *P*-values less than 0.05 were considered statistically significant.

## 3. Results

### 3.1. Huh7.5 Cell Line Overexpressing NTCP (Huh7.5-NTCP Cells)

**Figure 1:**
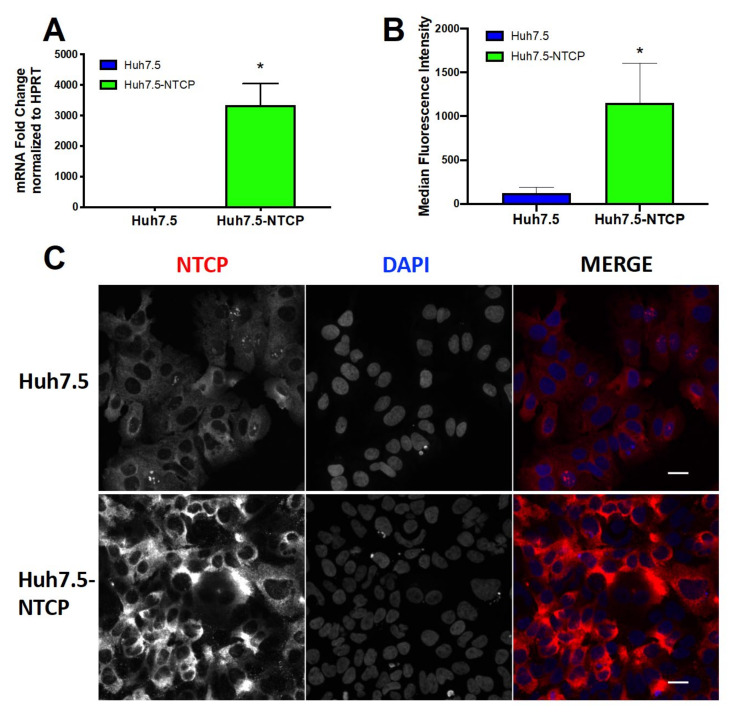
Overexpression of NTCP in Huh7.5 cells. (**A**) Lentiviral-transduced puromycin-selected Huh7.5-NTCP cell line expressed more NTCP mRNA than the parental Huh7.5 cell line. RT-qPCR was used to measure NTCP and hypoxanthine-guanine phosphoribosyltransferase (HPRT) mRNA levels. Huh7.5-NTCP cells expressed more cell surface NTCP than parental Huh7.5 cells as illustrated with (**B**) flow cytometry and (**C**) immunofluorescence (IF) microscopy. Immunofluorescent staining of NTCP is shown in red, and the DAPI (4′,6-diamidino-2-phenylindole) stain of nuclei is shown in blue. Images show a single plane/z-stack. The scale bars are 10 μm. (**A**,**B**) Average values with error bars (±SD) derived from three experiments are plotted. Unpaired Student’s *t*-test was used for statistical analysis. * *p* < 0.05; *n* = 3.

To establish an in vitro HBV infection model, we first subjected Huh7.5 cells to transduction with lentiviral NTCP expression constructs followed by puromycin selection. The resulting Huh.7.5-NTCP cell line had more than a 3500-fold increase in NTCP mRNA when compared with the parental Huh.7.5 cell line using real-time quantitative polymerase chain reaction (RT-qPCR) (Figure 1A). Flow cytometry analysis revealed increased cell surface expression of NTCP (Figure 1B) and immunofluorescent staining followed by confocal microscopy (Figure 1C) also showed increased expression of cell surface NTCP protein in Huh7.5-NTCP cells compared to the parental cell line.

### 3.2. Human Serum Culture Enhanced Productive HBV Infection in Huh7.5-NTCP Cells

We tested the permissiveness of Huh7.5-NTCP cells in human serum culture to HBV infection. Previous studies reported that NTCP-expressing HepG2 and AML12 cells were permissive to HBV infection and that treatment with DMSO significantly promoted HBV replication and production [41]. Cell culture protocols for HBV infection commonly use Dulbecco’s modified Eagle’s medium (DMEM) supplemented with 10% fetal bovine serum (FBS) and require 2–2.5% DMSO throughout. We compared HBV infection of Huh7.5-NTCP cells cultured in DMEM and supplemented either with FBS or human serum (HS) with or without the addition of DMSO.

**Figure 2:**
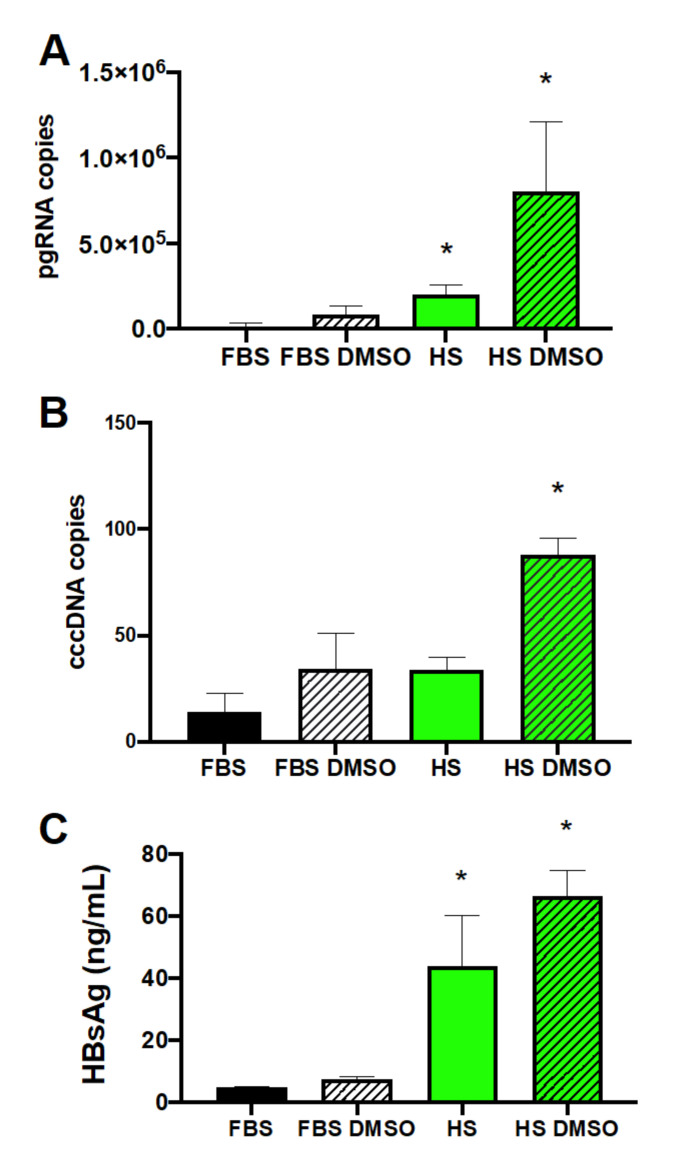
Enhancement of HBV replication by human serum culture. Human serum culture increased HBV (**A**) pgRNA, (**B**) cccDNA, and (**C**) HBV surface antigen (HBsAg) levels from Huh7.5-NTCP cells. Huh7.5-NTCP cells were cultured in the media supplemented with FBS or HS and with or without the addition of DMSO during HBV infection. Samples were collected on day 14 (**A**,**B**) or day 7 (**C**) post-infection. Pregenomic RNA was measured using RT-qPCR from 10 ng of total RNA. Covalently closed circular DNA was quantified using q-PCR from 10 ng of gDNA. HBsAg was measured in a culture supernatant using enzyme-linked immunosorbent assay (ELISA). Average values with error bars (±SD) derived from three experiments are plotted. One-way analysis of variance (ANOVA) was used with the Bonferroni correction for multiple comparison test. * *p* < 0.05.

We assayed HBV pregenomic RNA (pgRNA), covalently closed circular DNA (cccDNA), surface antigen (HBsAg), and E antigen (HBeAg). Analysis of HBV pgRNA by RT-qPCR 14 days after infection showed that Huh7.5-NTCP cells cultured in the HS-supplemented DMEM medium produced 12-fold more copies of pgRNA than the cells cultured in the standard FBS-supplemented DMEM medium (Figure 2A). The FBS-supplemented culture required DMSO (2%) during HBV infection, and the pgRNA level was 10-fold lower than that in the HS-supplemented cultures if DMSO was also added during HBV infection (Figure 2A). The earliest biochemical step in HBV infection is the generation of HBV cccDNA from which pgRNA is transcribed. Measured using qPCR, the cccDNA levels in the HBV-infected Huh7.5-NTCP cells were higher when cultured in the medium supplemented with HS than in the FBS culture conditions (Figure 2B).

HBsAg released into the supernatant of infected cells was measured using enzyme-linked immunosorbent assay (ELISA). The supernatants of HS-supplemented cultures had significantly higher levels of HBsAg than did the FBS-supplemented cultures with or without further supplementation of DMSO during infection (Figure 2C). Additional analysis of the secreted HBeAg (Figure S2) showed higher levels of HBV proteins in the HS-supplemented cultures than in the FBS-supplemented cultures. Together, these results of pgRNA, cccDNA, and HBV proteins all support the conclusion that Huh7.5-NTCP cells in cultures supplemented with human serum enhance HBV infection.

We also examined whether the effect of HS-supplemented culture on HBV infection of Huh7.5-NTCP cells was consistent at a lower multiplicity of infection (MOI). The levels of HBV pgRNA from cells infected with 100 genome equivalents per cell (Figure S4) showed similar trends to results from cells infected with 500 genome equivalents per cell (Figure 2A), with pgRNA levels increasing with HS supplementation compared to FBS-supplemented cultures. This shows that the enhancement of HBV infection of Huh7.5-NTCP cells using the HS-supplemented cultures is sustained at a lower MOI (Figure S4). Since the HS culture contains lower serum concentrations than the FBS culture, we tested culture conditions containing 10% FBS, 4% FBS, and 4% HS (Figure S5). While cells supplemented with 4% FBS contained higher levels of HBV pgRNA, they did not reach the levels in the cultures that were HS-supplemented (Figure S5). Thus, the observed enhancement of HBV infection is not due to a difference in the concentrations of serum in the culture medium.

This is the first report of a human serum culture system enhancing HBV infection of Huh7.5-NTCP cells as well as the first hepatoma cell culture HBV infection system that does not require DMSO. Our human serum differentiation of Huh7.5-NTCP hepatoma cell system offers an alternative in vitro tool for studying HBV infection. It complements primary human hepatocytes (PHHs), which are difficult to acquire and the only other in vitro model where DMSO is not required for HBV infection [52].

### 3.3. Huh7.5-NTCP Cells in a Human Serum Culture Serve as a Model for Long-Term HBV Infection

**Figure 3:**
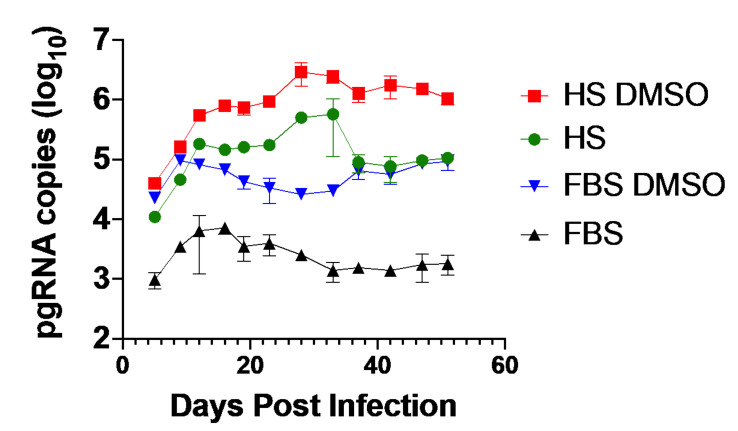
Sustained infection by HBV in Huh7.5-NTCP cells cultured in human serum. Huh7.5-NTCP cells cultured in the medium supplemented with FBS or HS were infected with 500 genome equivalents per cell with or without 2% DMSO during infection. HBV pgRNA in the cells was repeatedly measured using RT-qPCR every 4–5 days for 50 days after HBV infection. Average values with error bars (±SD) derived from three experiments are plotted.

To investigate whether Huh7.5-NTCP cells remained infected for a prolonged period and could potentially model chronic HBV infection, we analyzed RNA over a period of 50 days after HBV infection. HBV pgRNA increased for approximately two weeks after infection followed by a plateau and sustained pgRNA levels for the remainder of the experiment (Figure 3). The pgRNA levels were consistently higher in Huh7.5-NTCP cells cultured in the medium supplemented with HS with or without further supplementation of DMSO compared to cultures in the media supplemented with FBS in the presence or absence of DMSO. The pgRNA levels were consistently the highest in the HBV-infected cells cultured in the media supplemented with HS and DMSO throughout the 50-day post-infection period (Figure 3). These results suggest that the Huh7.5-NTCP cell line in the human serum culture system could potentially serve as an in vitro model for chronic HBV infection [52,58].

### 3.4. Human Serum Alters Hepatocyte Differentiation Markers in Huh7.5-NTCP

**Figure 4:**
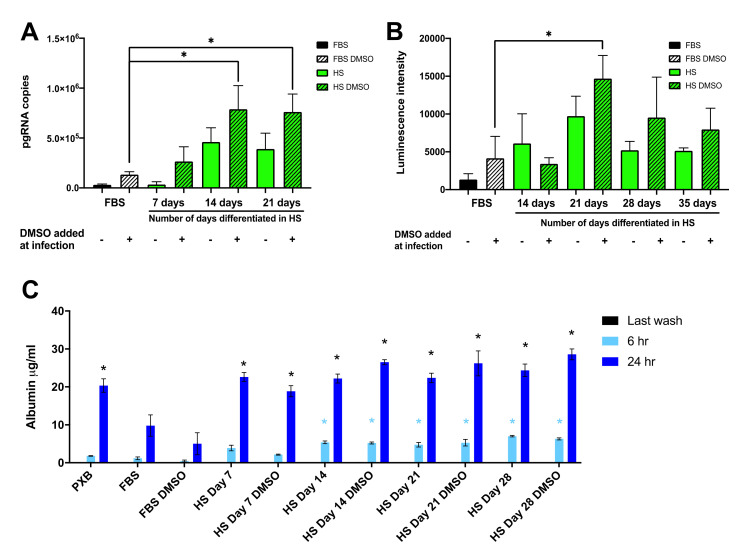
Enhancement of HBV replication and expression of hepatocyte markers in Huh7.5-NTCP cells cultured in human serum. (**A**–**C**) Huh7.5-NTCP cells were cultured for various lengths of time in a medium supplemented with FBS or HS. Cells maintained in HS-supplemented media were infected after the indicated number of days in HS-containing media. During HBV infection, DMSO was either absent (−) or present (+). Samples were collected on day 14 post-infection for (**A**) RT-qPCR analysis of pgRNA or (**B**) nanoluciferase reporter luminescence analysis. (**A**,**B**) One-way analysis of variance (ANOVA) was used with the Bonferroni correction for multiple comparison test. * *p* < 0.05. (**C**) Secreted human albumin concentration after 6 h and 24 h was determined using ELISA. Average values (±SD) derived from three experiments are plotted. Two-way analysis of variance (ANOVA) was used with the Bonferroni correction for multiple comparison test. Blue *, *p* < 0.01 compared to FBS albumin secretion in 6 h. Black *, *p* < 0.01 compared to FBS albumin secretion in 24 h.

We reasoned that enhancement of HBV infection of Huh7.5-NTCP cells in the HS-supplemented culture medium could be due to differentiation of Huh7.5-NTCP cells to become more hepatocyte-like. Previously we showed that 21 days were required to fully differentiate Huh7.5 cells in HS media [43,44]. We therefore tested the optimum time needed for the Huh7.5-NTCP cells to differentiate in the HS-supplemented culture medium. We cultured Huh7.5-NTCP cells in a medium containing HS for 7, 14, and 21 days prior to HBV infection of the cells. We then measured HBV pgRNA 14 days after HBV infection. The levels of HBV pgRNA increased with differentiation time prior to infection and reached a maximum between 14 and 21 days of differentiation in the HS-supplemented medium (Figure 4A).

We used the nanoluciferase recombinant virus and nanoluciferase luminescence assays as a surrogate marker for early steps in HBV infection [57]. Luminescence intensity was the highest when the cells were differentiated in the HS-supplemented medium for 21 days prior to HBV infection (Figure 4B). These results suggest that culturing in the HS-supplemented medium for 14 to 21 days prior to HBV infection is optimum for the enhanced HBV infection, which is consistent with our previous observations for the time required for HS-mediated differentiation and full restoration of hepatocyte functions [43,44].

Using ELISA, we assessed albumin secretion, which is a conventional marker of differentiation and viability of PHHs. Culturing Huh7.5-NTCP cells in the HS-supplemented medium increased to amounts approaching that produced by plated PHHs [59] and PXB cells (human hepatocytes isolated from chimeric humanized liver mice and then cultured in vitro) (Figure 4C). Albumin secretion increased during the initial seven days of the HS-supplemented cultures and this increased amount of albumin secretion was maintained throughout the entire 28 days of the HS-supplemented cultures (Figure 4C). These findings suggest that the culture in the HS-supplemented medium modified the Huh7.5-NTCP hepatoma cell line to a hepatocyte-like phenotype similar to the effect of HS-media on Huh7.5 cells [44,45,46], and this correlates with the enhanced HBV infection (Figure 2). The increase in hepatocyte differentiation markers suggest that the cells cultured in the HS-containing medium have more differentiated characteristics than the cells cultured in the standard FBS-containing medium. The HS-induced cell differentiation may be a factor in the ability of HBV to infect the cells and maintain production of pgRNA when cultured in the HS-containing medium.

### 3.5. Involvement of NTCP and Possible Effect of Its N-Glycosylation on Viral Entry

**Figure 5:**
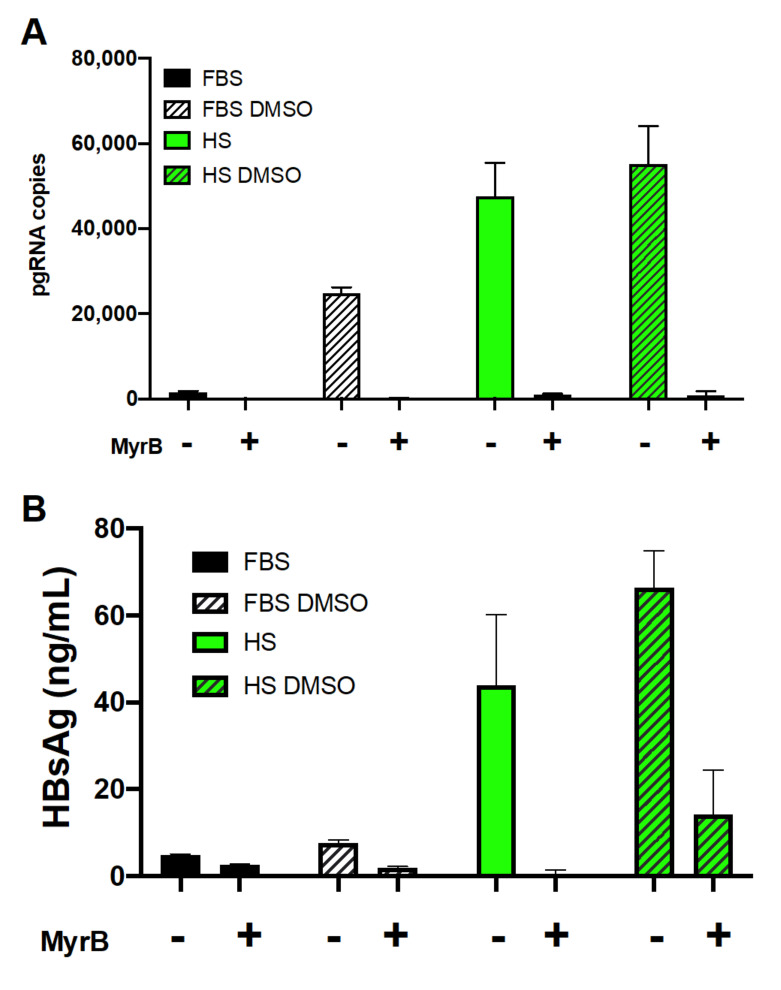
Reduction of HBV infection by MyrB, an entry inhibitor. MyrB was added to culture at 300 nM 30 min prior to infection and remained during HBV infection and one day post-infection. Cell monolayers and the culture supernatant were collected on day 7 post-infection for (**A**) RT-qPCR analysis of pgRNA and (**B**) ELISA of the surface antigen (HBsAg). Average values with error bars (±SD) derived from three experiments are plotted.

We investigated how the human serum culture system affected expression of NTCP, the putative HBV entry receptor. Administration of Myrcludex B (MyrB), a peptide mimic of the portion of the surface antigen that binds NTCP [60], inhibited infection of cells under all four culture conditions (Figure 5). This suggests that NTCP mediated HBV entry into these cells.

**Figure 6:**
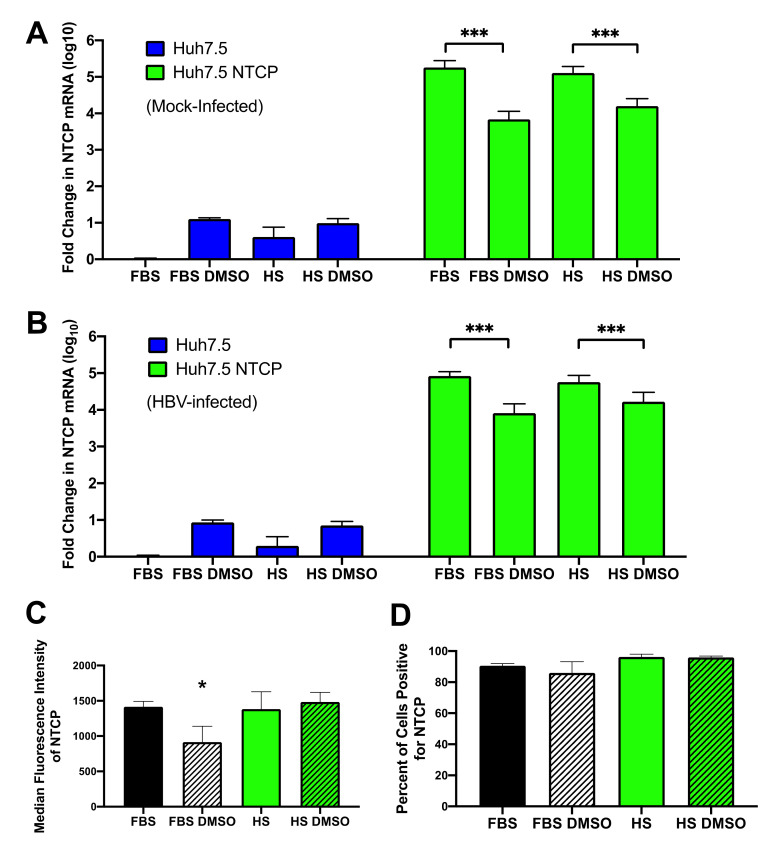
Changes in NTCP mRNA levels and surface NTCP protein expression under various culture conditions. Huh7.5 or Huh7.5-NTCP cells were (**A**) not infected with the virus (mock), or (**B**) infected with HBV. Samples were collected on day 7 post-infection for RT-qPCR analyses of NTCP mRNA and HPRT mRNA levels. ΔΔCT values were calculated to determine fold changes in NTCP mRNA expression normalized to that of the Huh7.5 cells cultured in the medium containing FBS. Huh7.5-NTCP cells were analyzed with flow cytometry to assess (**C**) cell surface expression of NTCP based on median fluorescence intensity and (**D**) the percentage of cells expressing NTCP. Average values with error bars (±SD) derived from three independent experiments are plotted. One-way analysis of variance (ANOVA) was used with the Bonferroni correction for multiple comparison test. * *p* < 0.05; *** *p* < 0.0005.

DMSO has been shown to increase HBV infection in cell cultures containing FBS, and this is consistent with what we see in Huh7.5 cells where DMSO increased NTCP expression (Figure 6). However, in Huh7.5 cells overexpressing NTCP, the addition of DMSO to either the FBS-supplemented cultures or the HS-supplemented cultures decreased the expression of NTCP mRNA levels (Figure 6A,B). Flow cytometry analyses of Huh7.5-NTCP cells indicated that levels of NTCP on the cell surface were lower when cells were cultured in the medium supplemented with both FBS and DMSO (Figure 6C). The decrease in NTCP levels caused by DMSO in NTCP-overexpressing cells was counterintuitive and led us to explore other possible reasons for the enhancement of HBV infection by HS and DMSO.

**Figure 7:**
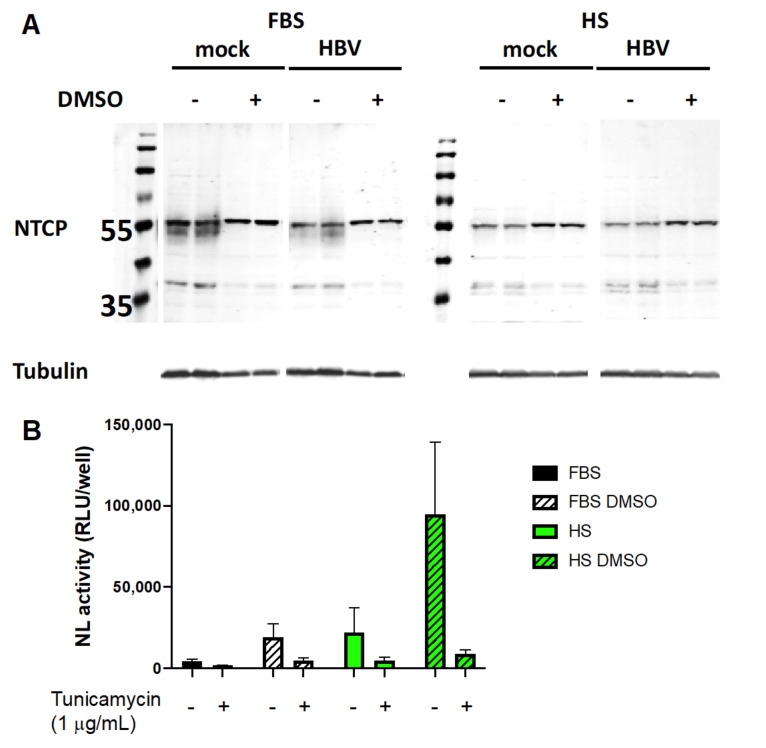
NTCP glycosylation and inhibition by tunicamycin. (**A**) Western blot analyses of NTCP glycosylation in Huh7.5-NTCP cells that were uninfected (mock) or infected with HBV. (**B**) Inhibition of N-glycosylation with tunicamycin suppressed HBV infection. Huh7.5-NTCP cells were incubated with 1 μg/mL tunicamycin for 2.5 h, followed by washing four times with PBS prior to infection. The cells were infected with nanoluciferase-expressing HBV (HBVNL) (MOI 500). Luminescence in relative light units (RLU) per well was measured to indicate nanoluciferase (NL) activity. Average values with error bars (±SD) derived from three experiments are plotted.

We examined the possible role of N-glycosylation of NTCP on viral entry. Western blots of lysates from Huh7.5-NTCP cells cultured in the absence of DMSO probed with NTCP-specific antibodies displayed two bands, one slightly above 35 kDa and one at 55 kDa (Figure 7A). Unglycosylated NTCP has a molecular weight of 37 kDa and the N-glycosylated form has a molecular weight of 55 kDa. The N-glycosylated form traffics to the cell surface and is required for HBV infection [61,62]. Western blot analyses of the cells cultured in the FBS-supplemented medium without DMSO exhibited a smear below the 55 kDa band, suggesting incomplete glycosylation of NTCP, while the cells cultured in the medium containing both FBS and DMSO had the sharp 55 kDa band, indicating full glycosylation. Western blots of lysates from Huh7.5-NTCP cells cultured in the HS-supplemented medium with or without supplementation of DMSO consistently had a sharp band at 55 kDa. The levels and species of NTCP do not change upon HBV infection of Huh7.5-NTCP cells (Figure 7A). These results suggest both that the decrease in NTCP mRNA levels caused by DMSO (Figure 6A,B) and that enhanced HBV infection of Huh7.5-NTCP cells in HS-supplemented cultures may in part be due to increased expression of fully glycosylated NTCP on the surface of Huh7.5-NTCP cells. Similarly, DMSO improves glycosylation of NTCP in Huh7.5-NTCP cells cultured in FBS and this treatment likely increases their infectability by HBV.

To explore whether glycosylation of NTCP was involved, we treated Huh7.5-NTCP cells in various culture media with tunicamycin [63], an N-glycosylation inhibitor, for 2.5 h prior to infection with HBV. We used a non-replicative nanoluciferase-expressing HBV (HBVNL) (Figure S3) to assess whether the tunicamycin treatment affected viral entry and early steps in HBV infection. Treatment with tunicamycin under all four culture conditions resulted in marked reductions in nanoluciferase activity (Figure 7B). Because the nanoluciferase activity of the cells infected with HBV containing the nanoluciferase reporter recapitulates only early events of infection, the suppression of infection by an inhibitor of N-glycosylation suggests N-glycosylation of NTCP is relevant to viral entry. Therefore, the full N-glycosylation of NTCP observed from Huh7.5-NTCP cells either cultured with HS- or DMSO-containing media may aid in the entry step of HBV infection.

## 4. Discussion

This report describes the first robust hepatoma cell culture HBV infection system that does not require DMSO. The only previous example in which DMSO was not required for HBV infection used primary human hepatocytes (PHHs) [52]. Because primary human hepatocytes are more difficult to acquire, our human serum culture of the Huh7.5-NTCP hepatoma cell system offers an alternative in vitro model for studying HBV infection.

It has been recognized that the more differentiated a liver cell culture model is, the more likely the culture system is permissive and supportive of HBV infection [14,22,25,26,52]. With actively dividing hepatoma cell lines and ex vivo primary hepatocyte cultures, differentiated phenotypes are conventionally established and maintained with the addition of DMSO to the culture media. The DMSO supplementation causes growth arrest and more hepatocyte-like gene expression profiles in hepatoma cell lines. However, DMSO causes cytotoxicity with its solvent properties [47,48,49,50,51] and fails to restore many liver functions in hepatoma cultures [43]. In both hepatic and cardiac tissue types (3D microtissue cultures) exposed to 0.1% DMSO, “transcriptome analysis detected >2000 differentially expressed genes affecting similar biological processes, indicating consistent cross-organ actions of DMSO” [51].

Previous studies in our laboratory showed widespread changes in gene expression when Huh7.5 cells are cultured in HS, shifting toward a phenotype more resembling PHHs [43,44]. Likewise, after transduction and overexpression of NTCP, the Huh7.5-NTCP cells exhibited contact inhibition and growth arrest when cultured in the HS-supplemented medium and retained these properties. Albumin secretion, a conventional marker of PHH function, showed that Huh7.5-NTCP cells also acquired a more differentiated phenotype in the HS-supplemented cultures.

This better differentiation induced by HS culture compared to the conventional FBS cultures may be attributed to the different growth factors, differentiation factors, and lipid composition of HS compared to FBS. Given the complex composition of human serum, empirical testing of specific differentiation factors is challenging and unlikely to reveal individual causative agents responsible for the 22–32% transcriptional changes observed with HS supplementation [44]. Although DMSO can induce growth arrest and increased transcription of some hepatocyte genes, it does not cause the comprehensive phenotypic shift towards primary liver characteristics brought about by HS-supplemented cultures. Therefore, this considerable restoration of liver function and metabolism by culture in human serum likely contributes to the observed enhancement of HBV infection and holds benefits over DMSO supplementation for physiologically relevant in vitro studies of HBV.

Future research may assess whether human serum culture can enhance the permissiveness of other HBV infection models, e.g., HepG2-NTCP cells or PHHs. Indeed, the HepG2-NTCP hepatoma cell line is commonly used for in vitro HBV studies because it is more permissive to HBV infection than Huh7 or Huh7.5 cells [36]. In Huh7.5-NTCP cells, HS differentiation promotes a more hepatocyte-like phenotype and significantly enhances HBV infection. However, the pgRNA level in HBV-infected and HS-differentiated Huh7.5-NTCP cells (Figure 2A) is lower than that in HepG2-NTCP cells (Figure S6). Our preliminary results (Figure S6) show that HepG2-NTCP cells require DMSO for HBV infection and can be infected in the presence of human serum and DMSO. It would be useful to test whether HepG2-NTCP cells differentiate or PHHs remain differentiated in human serum, and if so, optimize this differentiation protocol. Figure 4A shows that Huh7.5-NTCP cells need to differentiate in human serum for 21 days before enhanced HBV infection is achieved. Likely because the HepG2-NTCP cells were only cultured short-term in a medium with human serum, there was no enhancement of HBV infection. Enhanced infection of HepG2-NTCP cells might not occur until these cells are differentiated in human serum.

We examined how culturing Huh7.5-NTCP cells with various media affected NTCP. Culture with DMSO supplementation resulted in reduced NTCP mRNA levels. Among the various culture media, cells cultured with FBS and DMSO supplementation displayed reduced surface protein expression of NTCP. N-glycosylation of NTCP was promoted in culture media supplemented with HS or DMSO. The inhibition of N-glycosylation suppressed HBV infection. Our results showing this potential involvement of NTCP N-glycosylation in HBV entry are consistent with those previously reported [61,62], although another study deemed this NTCP modification non-essential to HBV infection [63].

This work could be extended by further studies of NTCP glycosylation and its impact on viral entry. To evaluate the contribution of NTCP glycosylation to the HS phenotype, future research could be conducted by mutating NTCP glycosylation sites (e.g., N5Q and N11Q) [61,63], transducing Huh7.5 cells with the NTCP mutants, and evaluating whether culture in HS leads to an increase in HBV replication.

## Data Availability

Data sharing is not applicable to this article.

## Supplementary Materials

The following are available online at https://www.mdpi.com/1999-4915/13/1/97/s1, Figure S1: Timeline for culturing and infecting Huh7.5-NTCP cells; Figure S2: Aptamer-binding assay for the E antigen of HBV (HBeAg); Figure S3: Pregenomic RNA and open reading frames derived from wild-type HBV, the plasmids pHBVNL and HBV-D; Figure S4: HBV pgRNA levels in Huh7.5-NTCP cells infected with a multiplicity of infections (MOI) of 100 genome equivalents per cell; Figure S5: HBV pgRNA levels in infected Huh7.5-NTCP cells that were cultured under different conditions; Figure S6: HBV pgRNA levels in infected HepG2-NTCP cells that were cultured and infected under different conditions.
