# Supplementary material for: In Vitro Infection with Hepatitis B Virus Using Differentiated Human Serum Culture of Huh7.5-NTCP Cells without Requiring Dimethyl Sulfoxide
Source: Viruses. 2021 Jan 12;13(1):97. doi: 10.3390/v13010097 (PMC7828204; doi:10.3390/v13010097)
Supplement: Supplementary file 1 [file viruses-13-00097-s001.pdf]

Supplementary Materials

In vitro infection with hepatitis B virus using differentiated human serum culture of Huh7.5-NTCP cells without requiring dimethyl sulfoxide

Connie Le, Reshma Sirajee, Rineke Steenbergen, Michael A. Joyce, William R. Addison, D. Lorne Tyrrell *

Li Ka Shing Institute of Virology, Department of Medical Microbiology and Immunology, 6010 Katz Centre for Health Research, University of Alberta, Edmonton, Alberta, Canada T6G 2E1

*Corresponding author.

Telephone: 1-780-492-8415

Fax: 1-780-492-5304

E-mail: lorne.tyrrell@ualberta.ca

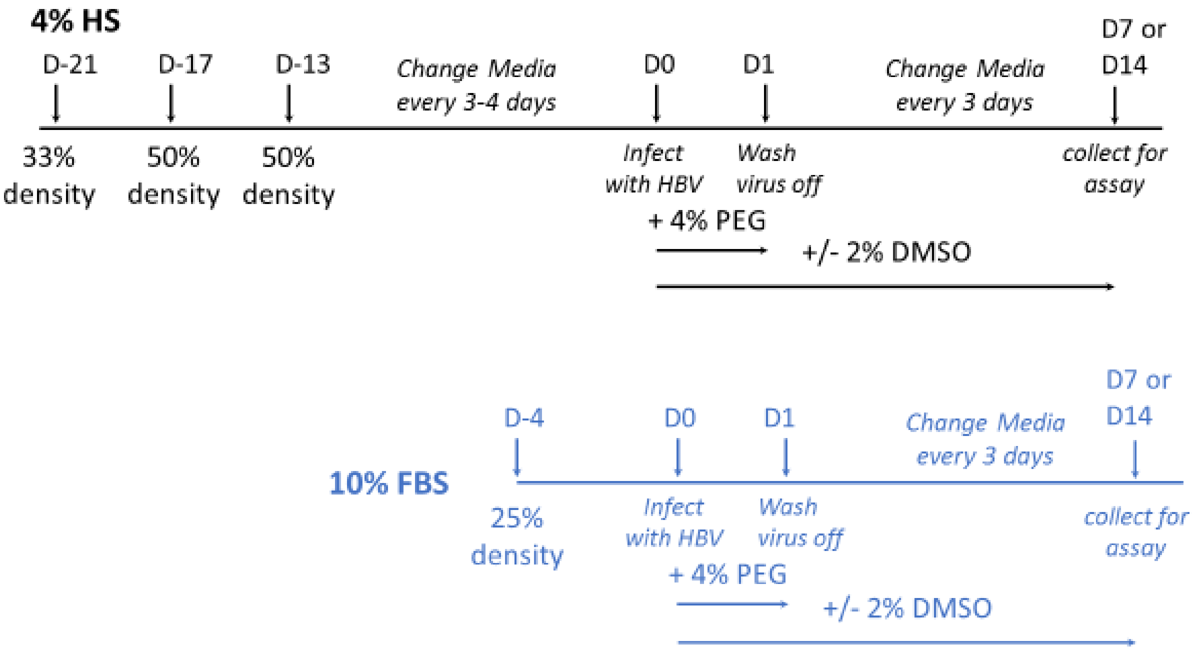

Figure S1. Schematic illustrating the timeline for culturing and infecting Huh7.5 NTCP cells. The day of inoculation with HBV is denoted as day 0 (D0). Days prior to infection are labelled with negative numbers (e.g., 21 days prior to infection is D-21). The percent density describes the cell seeding density onto new flasks or plates on the indicated day. Infection was done by HBV inoculation overnight along with 4% polyethylene glycol (PEG) 8000 and with or without 2% DMSO supplementation. The PEG was removed from cultures after infection. The infected cells were maintained in DMEM medium supplemented with FBS or HS and with or without 2% DMSO supplementation.

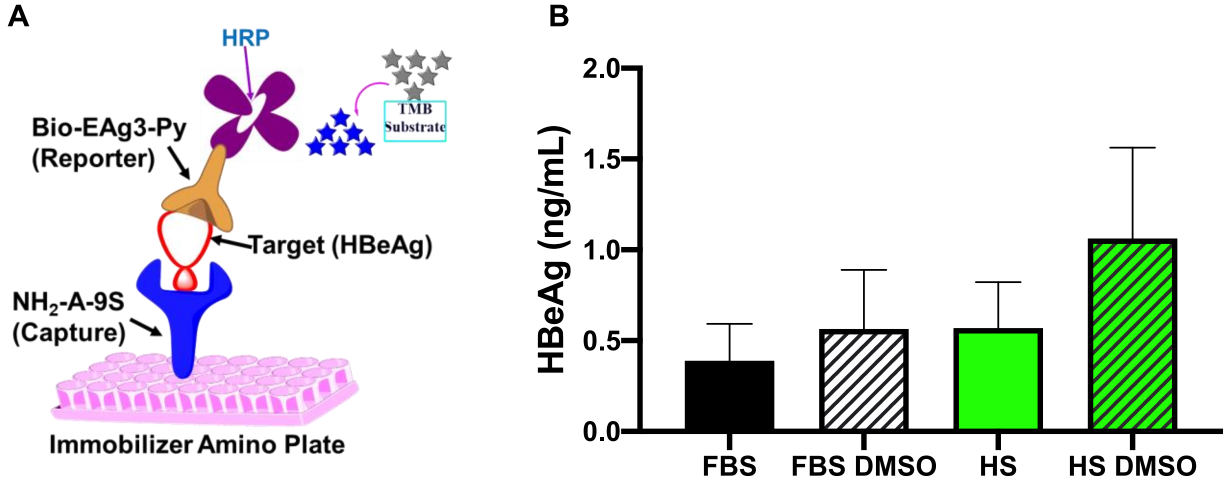

Figure S2. (**A**) Schematic showing the aptamer binding assay for the E antigen of HBV (HBeAg). (**B**) Secreted E antigen in HBV infected Huh7.5 NTCP cells. Huh7.5-NTCP cells were cultured in DMEM medium supplemented with FBS or HS. Cells were infected with multiplicity of infections (MOI) of 500 genome equivalents per cell. In a parallel set of experiments, 2% dimethyl sulfoxide (DMSO) was added during and after HBV infection. Culture supernatant was collected on day 7 post infection for the quantification of HBeAg using the aptamer binding assay. Average values with error bars (±SD) derived from three experiments are plotted.

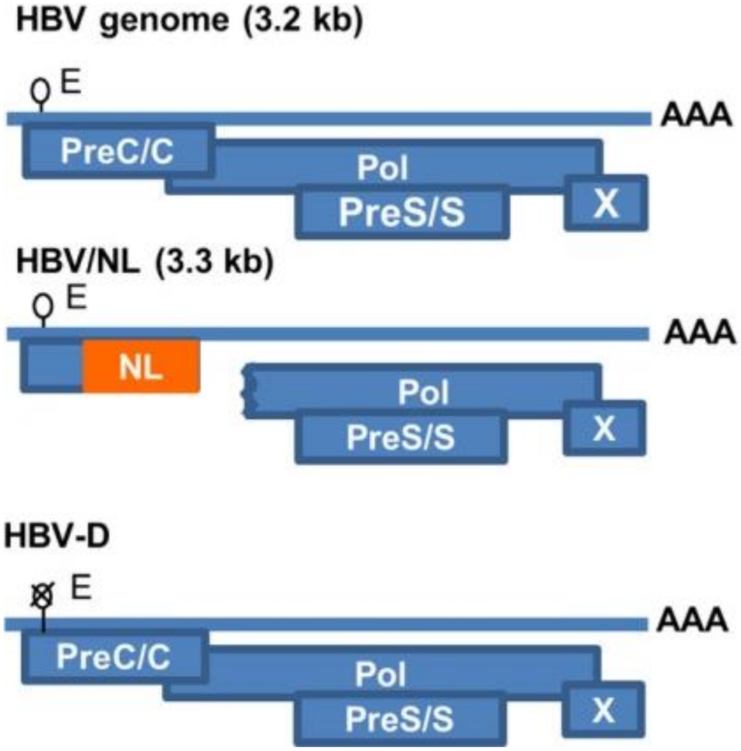

Figure S3. Depiction of pregenomic RNA (pgRNA) and open reading frames derived from wildtype HBV, the plasmids pHBVNL, and HBV-D. PreC/C denotes precore/core; Pol is polymerase; PreS/S is pre-surface/surface antigen; X is the X protein; and NL is nanoluciferase. The "AAA" represents the poly A tails at the 3'-end of the pgRNA. The stem loop labelled with "E" denotes the epsilon sequence required for packaging of the pgRNA. The HBV-D "E" stem loop filled with "x" represents mutations leading to a defect in secondary structure formation and encapsidation of the HBV-D pregenome. Modified from Nishitsuji, *et al.* Novel reporter system to monitor early stages of the hepatitis B virus life cycle. *Cancer Sci.* **2015**, 106, 1616-1624. doi: 10.1111/cas.12799.

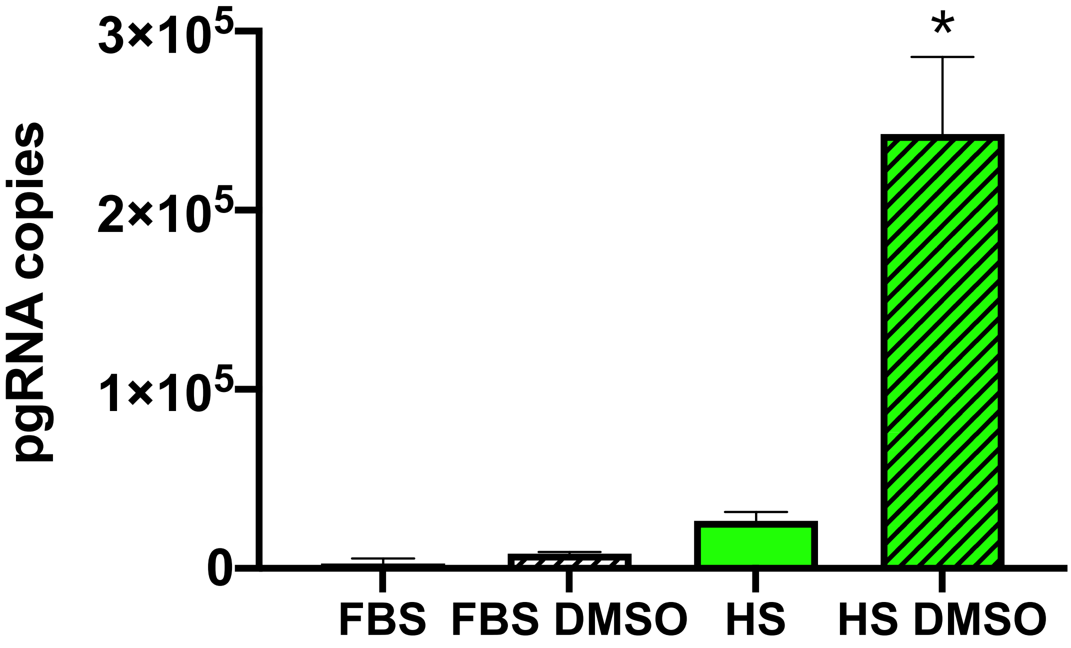

Figure S4. HBV pgRNA levels in Huh7.5 NTCP cells infected with a multiplicity of infections (MOI) of 100 genome equivalents per cell. Huh7.5-NTCP cells were cultured in DMEM medium supplemented with FBS or HS. In a parallel set of experiments, 2% DMSO was added during and after HBV infection. Samples were collected on day 14 post infection for RT-qPCR analysis of HBV pgRNA from 10 ng total RNA. One-way analysis of variance (ANOVA) was used with Bonferroni's correction for multiple-comparison test. *, $P<0.05$ compared to the FBS culture condition; n=3. The error bars indicate standard deviation.

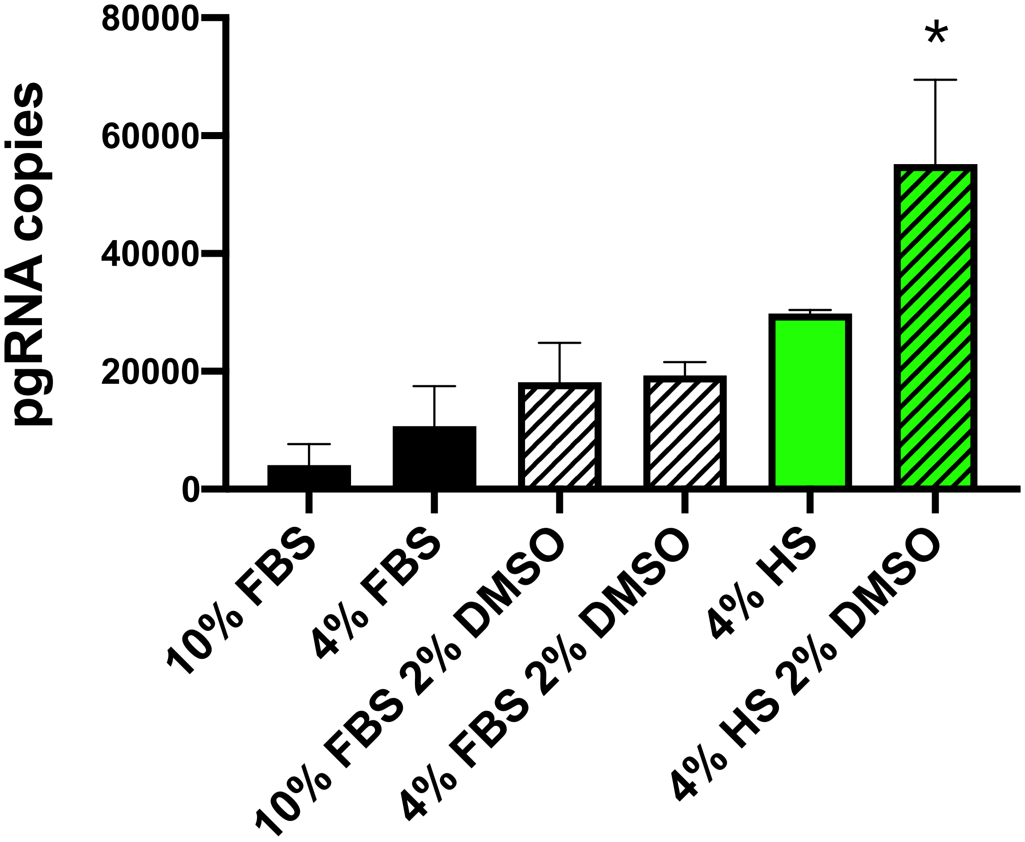

Figure S5. HBV pgRNA levels in infected Huh7.5 NTCP cells that were cultured under different conditions. Huh7.5-NTCP cells were cultured in DMEM medium supplemented with 10% FBS, 4% FBS, or 4% HS. In a parallel set of experiments, 2% DMSO was added during and after HBV infection. Cells were infected with multiplicity of infections (MOI) of 500 genome equivalents per cell. Samples were collected on day 14 post infection for RT-qPCR analysis of pgRNA from 10 ng total RNA. One-way analysis of variance (ANOVA) was used with Bonferroni's correction for multiple-comparison test. *, $P<0.05$; n=3. The error bars indicate standard deviation.

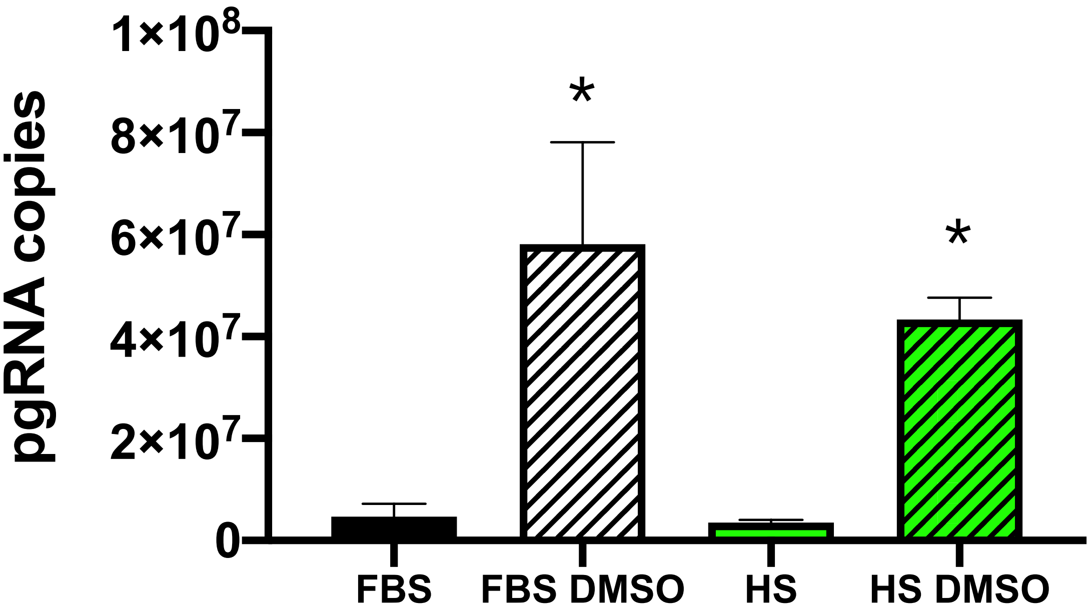

Figure S6. HBV pgRNA levels in infected HepG2-NTCP cells that were infected and cultured in different medium. HepG2-NTCP cells were infected and cultured in DMEM medium supplemented with 10% FBS, 10% FBS and 2% DMSO, 4% HS, or 4% HS and 2% DMSO. Cells were infected with multiplicity of infections (MOI) of 1000 genome equivalents per cell. Samples were collected on day 7 post infection for RT-qPCR analysis of pgRNA from 10 ng total RNA. One-way analysis of variance (ANOVA) was used with Bonferroni's correction for multiple-comparison test. *, $P<0.05$ comparing FBS DMSO to FBS as well as comparing HS DMSO to HS conditions; n=3. The error bars indicate standard deviation.
